# *Angiostrongylus cantonensis* DNA in Cerebrospinal Fluid of Persons with Eosinophilic Meningitis, Laos

**DOI:** 10.3201/eid2312.171107

**Published:** 2017-12

**Authors:** Damien K.Y. Ming, Sayaphet Rattanavong, Tehmina Bharucha, Onanong Sengvilaipaseuth, Audrey Dubot-Pérès, Paul N. Newton, Matthew T. Robinson

**Affiliations:** Imperial College London, London, UK (D.K.Y. Ming);; Lao-Oxford-Mahosot Hospital Wellcome Trust Research Unit, Vientiane, Laos (D.K.Y. Ming, S. Rattanavong, T. Bharucha, O. Sengvilaipaseuth, A. Dubot-Pérès, P.N. Newton, M.T. Robinson);; University College London, London (T. Bharucha);; Aix-Marseille Université, Marseille, France (A. Dubot-Pérès);; University of Oxford, Oxford, UK (P.N. Newton, M.T. Robinson)

**Keywords:** *Angiostrongylus cantonensis*, eosinophilic meningitis, meningitis/encephalitis, parasites, cerebrospinal fluid, central nervous system infections, quantitative PCR, DNA, Laos, Lao Peoples’ Democratic Republic

## Abstract

Definitive identification of *Angiostrongylus cantonensis* parasites from clinical specimens is difficult. As a result, regional epidemiology and burden are poorly characterized. To ascertain presence of this parasite in patients in Laos with eosinophilic meningitis, we performed quantitative PCRs on 36 cerebrospinal fluid samples; 4 positive samples confirmed the parasite’s presence.

Humans are incidental hosts of *Angiostrongylus cantonensis* nematodes; global distribution of these nematodes is being increasingly recognized (*1*). Ingestion of larvae from undercooked infected snails or food contaminated with mollusk secretions can result in the migration of *A. cantonensis* parasites through the human central nervous system (CNS) (*2*). The presence of the parasite and associated inflammation in the CNS can contribute to a meningoencephalitic syndrome, typified by a cerebrospinal fluid (CSF) eosinophilia constituting >10% of total CSF leukocyte count. Formal diagnosis of angiostrongyliasis is difficult because the parasite is typically present in low numbers in the CSF (*3*). Serologic methods are limited by cross-reactivity with other helminths (*4*), and antibody-based methods may lack sensitivity, especially during acute illness (*5*).

Host sampling studies have identified *A. cantonensis* parasites in some Mekong region countries but not in Lao People’s Democratic Republic (Laos) (*6*). To ascertain the presence of this parasite in patients with eosinophilic meningitis in Laos, we tested samples from a cohort of 1,065 patients suspected of having CNS infection at Mahosot Hospital, Vientiane, Laos, during 2003–2013 by Giemsa staining and identified 54 CSF samples containing >10% eosinophils. Of these, 36 samples from 35 patients were available for this study (1 patient underwent lumbar puncture twice) and were tested by conventional and quantitative PCR (cPCR and qPCR). From the same cohort, we also performed qPCR testing on another 50 CSF samples with 1%–9% eosinophils.

DNA was extracted from 200 μL of CSF by using a QIAamp DNA Mini Kit (QIAGEN, Hilden, Germany) and eluted in 30 μL buffer. We ran the extract in parallel with positive samples (*A. cantonensis* DNA from experimentally infected rats, University of Sydney, Sydney, Australia) and negative controls by using published cPCR (*7*) and qPCR (*8*) protocols. We used Platinum PCR SuperMix (Thermo Fisher, Waltham, MA, USA) and performed assays on a Bio-Rad CFX96 (Bio-Rad, Watford, UK).

Among patients with CSF eosinophilia >10%, male patients predominated, although sex of patients did not differ significantly among patients with or without CSF eosinophilia (Table). Of the 36 CSF samples that contained >10% eosinophils, all were negative by cPCR, but 4 (11.1%) were positive for *A. cantonensis* DNA by qPCR; median quantification cycle was 35.9 (range 34.1–37.4). Sensitivity of qPCR was apparently higher than that of cPCR. The median duration of illness for patients with positive qPCR was 4 (range 0–10) days. Of 3 patients with a positive qPCR, 2 reported that they had eaten raw snails in the previous month.

**Table:** Demographics and clinical characteristics of 35 patients with eosinophilic meningitis tested by qPCR for *Angiostrongylus cantonensis* parasites*

Characteristic	Positive, n = 4†	Negative, n = 31†
Median age, y (IQR)	25 (21–42)	25 (20–40)
Sex, no. (%)		
M	4/4 (100)	19/31 (61)
F	0	12/31 (39)
Median duration of illness, d (IQR)	4 (1–9)	4 (0–6.5)
Median eosinophil % of total leukocyte count (IQR)	65 (50–73)	33 (14–56)
Diet in past mo		
Raw fish	3/3	7/13
Raw shellfish	2/2	3/11
Raw snails	2/3	5/12
Raw crab	2/2	2/11
Fulfilled WHO criteria for meningitis at admission, no. (%)	3/4 (75)	9/28 (32)
Median CSF opening pressure, cm H_2_O (IQR)	28 (22–35)	27 (17–40)
Median CSF leukocyte count, × 10^6^ cells/L (IQR)	583 (493–1,198)	125 (40–580)
Median CSF protein, g/L (IQR)	0.68 (0.31–1.08)	0.56 (0.31–1.00)
Median CSF glucose, mmol/L (IQR)	2.7 (2.4–4.2)	3.1 (2.4–4.9)

*CSF, cerebrospinal fluid; IQR, interquartile range; qPCR, quantitative PCR; WHO, World Health Organization. †Denominators vary according to available data.

Results from 2 CSF samples obtained from the same patient and tested by qPCR were discordant; CSF obtained after 7 days of illness was negative for *A. cantonensis* DNA, but a sample obtained on day 13 was positive (quantification cycle 34.1). This finding is consistent with previous observations (*8*), and it is plausible that during the acute stages of infection, insufficient nucleic material is present for detection. Although lumbar puncture is invasive, a high clinical suspicion of angiostrongyliasis in the context of a negative qPCR may therefore warrant a repeated lumbar puncture. All positive samples had CSF eosinophil proportions >40%, and all samples containing a 1%–9% eosinophil proportion tested negative by qPCR, supporting the conventional cutoff of a CSF eosinophilia >10% in the diagnosis of CNS angiostrongyliasis.

Our findings are consistent with those from studies demonstrating *A. cantonensis* parasites as a cause of eosinophilic meningitis in the region, although the proportion of *A. cantonensis*–positive cases in our cohort was lower than that from Vietnam (11.1% vs. 67.3%) (*9*). Results may be affected by factors such as geographic location, differences in healthcare access, and the contribution of other causes of eosinophilic meningitis, such as *Gnathostoma spinigerum* nematodes. In the absence of a reliable reference diagnostic standard, we were unable to estimate the proportion of false-negative results in this cohort or to correlate findings with other immunodiagnostic modalities. Further studies on eosinophilic meningitis from a wider geographic area and improved diagnostics would help establish the overall clinical burden of CNS angiostrongyliasis in Laos.

Diet (consumption of raw snails and food contaminated by snails) is considered a major risk factor for angiostrongyliasis, making an epidemiologic link and public health interventions a possibility. The exact host species responsible for transmission remains unclear, however, given that *A. cantonensis* parasites have apparently yet to be identified within snails in Laos.

Our identification of *A. cantonensis* DNA by qPCR of CSF samples from 4 (11%) of 35 patients with eosinophilic meningitis confirms the parasite’s presence in Laos. Further regional characterization, coupled with dietary surveys, will be invaluable for stratifying the risk for human transmission and guiding public health interventions.
